# How does temperature affect functional kleptoplasty? Comparing populations of the solar-powered sister-species *Elysia timida* Risso, 1818 and *Elysia cornigera* Nuttall, 1989 (Gastropoda: Sacoglossa)

**DOI:** 10.1186/s12983-018-0264-y

**Published:** 2018-04-24

**Authors:** Elise Marie Jerschabek Laetz, Heike Wägele

**Affiliations:** 1 Zoological Research Museum Alexander Koenig, 160 Adenauerallee, 53113 Bonn, Germany; 2 Institute for Evolutionary Biology and Ecology, University of Bonn, An der Immenburg 1, 53121 Bonn, Germany

**Keywords:** Kleptoplasty, *Elysia*, Intracellular digestion, Endosymbiosis, Sacoglossa

## Abstract

**Background:**

Despite widespread interest in solar-powered sea slugs (Sacoglossa: Gastropoda), relatively little is know about how they actually perform functional kleptoplasty. Sister-taxa *Elysia timida* and *E. cornigera* provide an ideal model system for investigating this phenomenon, since they feed on the same algal genus and only *E. timida* is capable of long-term kleptoplasty. Recent research has explored factors regarding functional kleptoplasty in *E. timida*, including their starvation longevity, digestive activity, autophagal response and photosynthetic efficiency under two different temperature conditions (18 °C and 21 °C). These studies revealed the trends *E. timida* displays regarding each factor during starvation as well as influences temperature has on some aspects of functional kleptoplasty. This study examines *E. cornigera* regarding each of these factors in an attempt to elucidate differences between each species that could explain their differing kleptoplastic abilities. Since both species naturally occur in 25 °C seawater (*E. timida* peak summer temperature, *E. cornigera* low winter temperature), each species was acclimatized to 25 °C to facilitate comparison and determine if these species exhibit physiological differences to starvation when under the same environmental conditions.

**Results:**

When comparing the different *E. timida* temperature treatments, it becomes clear that increased temperatures compromise *E. timida*’s kleptoplastic abilities. Specimens acclimatized to 25 °C revealed shorter starvation longevities surviving an average 42.4 days compared to the 95.9 day average observed in specimens exposed to 18 °C. Each temperature treatment displayed a significantly different decrease throughout the starvation period in both, the rate of photosynthetic efficiency and in the decreasing functional kleptoplast abundance. Lysosomal abundances are assessed here as indicators of different aspects of metabolic activity, which could be correlated to temperature. *E. cornigera,* also acclimatized to 25 °C did not display significantly similar patterns as any of the *E. timida* temperature treatments, having fewer incorporated kleptoplasts, a higher lysosomal response to starvation, a faster decrease in photosynthetic efficiency and a lower starvation longevity.

**Conclusions:**

These results confirm that each species has different physiological reactions to starvation and kleptoplast retention, even under the same conditions. While temperature affects aspects of functional kleptoplasty, it is likely not responsible for the differences in kleptoplastic abilities seen in these species.

**Electronic supplementary material:**

The online version of this article (10.1186/s12983-018-0264-y) contains supplementary material, which is available to authorized users.

## Background

Some members of the enigmatic sea slug clade, Sacoglossa (Gastropoda: Heterobranchia) are the only known metazoan taxa capable of enslaving functional chloroplasts from their algal food in a process entitled functional kleptoplasty [1–5]. *Elysia timida* Risso, 1818 has long been used as a model organism in investigations regarding functional kleptoplasty due to the extended period they can remain alive without access to food (averaging about 90 days) and their widespread distribution in high densities across the Mediterranean Sea [6–16]. The high functionality of their sequestered chloroplasts and the duration these kleptoplasts remain active, as revealed by Pulse Amplitude Modulated Fluorometry (PAM) data, groups *E. timida* with a few other species as a Long-term (plastid) Retaining – LtR species [7, 17–22].

LtR species are found throughout the world’s temperate and tropical oceans. *E. chlorotica* is probably the most well-known sacoglossan slug and holds the record for chloroplast retention with up to 14 months, depending on the report [17, 23–28]. This stenophagous slug, feeding only on *Vaucheria litorea* Agardh, 1873, is found on the western Atlantic coast, between Nova Scotia, Canada and Florida, USA [25]. *Plakobranchus ocellatus* Hasselt, 1824 is found throughout the Indo-Pacific where it feeds on numerous food algae and can survive starvation for up to 10 months, [23, 29–31]. *E. crispata* Mörch, 1863 and *E. clarki* Pierce, Curtis, Massey, Bass, Karl & Finney, 2006*,* currently debated as to whether they are two distinct species or one species with two morphotypes living in different ecosystems, are found in the Caribbean where they eat a multitude of algal species and last up to 40 days in starvation [17, 21, 22, 32–34]. The limapontioidean species *Costasiella ocellifera* (Simroth, 1895) also survives over 50 days [19]. *E. viridis* has been attributed to the LtR group, however reports on its starvation longevity and photosynthetic efficiency vary, leading some to remove it from this group [35–40]. Another species, *E. asbecki* Wägele, Stemmer, Burghardt, Händeler, 2010 may also retain functional kleptoplasts for extended durations, however this is only suggested due to PAM activity in the first few weeks of starvation and requires confirmation before it can be considered a LtR form [41].

Recent phylogenetic analyses have confirmed *E. timida* and *E. cornigera* Nutall, 1989 as sister-species [17, 33, 34, 42, 43]. These organisms are anatomically very similar, and were even synonymized as one species [44] before the name *E. cornigera* was resurrected and reassigned to the Caribbean populations, limiting *E. timida* to the Eastern Atlantic and Mediterranean Sea populations [42]. Although they are again considered separate species, *E. timida* and *E. cornigera* occur in similar habitats – coastal lagoons and the sublittoral zone at 0–10 m depth. Both species feed by sucking the cell contents out of *Acetabularia* sp. (Dasyclades: Chlorophyta) cells: *E. timida* feeds on the Mediterranean algal species *A. acetabulum* Silva, 1952*,* while *E. cornigera* feeds on Caribbean *Acetabularia* species [33]. Despite not encountering it in the field, *E. cornigera* is capable of feeding on *A. acetabulum* and has not been reported to have a diminished longevity when the food is switched [8, 45]. Interest in *E. timida* and *E. cornigera* as a model system is beginning to gain a foothold because *E. cornigera* cannot withstand the extended starvation periods *E. timida* can [45, 46]. While *E. timida* has a slow decline in photosynthetic efficiency throughout its long starvation period, *E. cornigera* kleptoplasts also begin high in efficiency, but then drop rapidly as the animal starves, leading to their classification as a Short-term Retaining (StR) form [33].

Populations of *E. timida and E. cornigera* have been recorded living in a range of differing types of habitats. Two previously recorded habitats for both species comprise coastal lagoons, characterized by highly fluctuating abiotic factors such as light, temperature and salinity [15, 42] and shallow sea areas, which provide a more stable environment, having less abiotic factor fluctuation due to depth and the consistent influx of new seawater. Despite the relative stability of shallow sea areas in contrast to lagoons, abiotic factors in shallow sea Mediterranean areas differ significantly from those in the Caribbean. Mediterranean salinity is higher with an average 37.8–38.6 practical salinity units (psu) in Blanes, Spain, whereas Caribbean seawater averages 35.5–36.5 psu (NOAA). *E. cornigera* is also naturally found in warmer waters than *E. timida*: the Caribbean surface temperature along the Florida Keys ranges between 25 and 30 °C each year [47], while the Mediterranean ranges from 14 to 25 °C at Blanes, Spain [48]. The range in temperatures *E. timida* naturally experiences has already been shown to influence a number of factors related to functional kleptoplasty [8]. Despite both species naturally occurring at 25 °C in shallow sea areas, these species have never been investigated under the same conditions with regard to functional kleptoplasty and their ability to withstand extended starvation.

Digestive activity and starch production in *E. timida* have been previously assessed for two different populations, each of which displayed significantly different results [37, 49]. A spring population was kept in a warmer environment (20–22 °C) reflecting the surface temperature where they were collected, while the fall population was collected and cultured at 18 °C matching the temperature from which they were collected. The trends observed in each of these investigations were the same, but the time frames differed, with the warmer population showing increases in digestive activity, starch maxima and death occurring earlier in the starvation period. This revealed that temperature may be responsible for this shift and that its effects warrant further study [49]. Additionally, the increased temperatures and photosynthetic rates almost align with published *E. cornigera* data, leading us to hypothesize that the lower temperatures naturally experienced by *E. timida* may facilitate long-term plastid retention and a long survival duration in starvation.

In this study, the following factors were compared in order to elucidate differences between these species and determine which factors are influenced by temperature: longevity in starvation, photosynthetic efficiency (PAM), functional kleptoplast abundance, digestive activity (lysosome activity within the digestive gland), autophagy (lysosomal activity outside the digestive gland) and excrement content for *E. cornigera* and *E. timida*. This is the first report to examine functional kleptoplast abundance, digestive activity, and excrement content for *E. cornigera*. *E. timida* specimens were compared under three temperature regimes reflecting natural conditions (18 °C, 21 °C, and 25 °C) to elucidate the effect temperature has on metabolic processes regarding functional kleptoplasty in this species. Furthermore, the 25 °C *E. timida* population reflects natural conditions this species experiences and mirrors those experienced by *E. cornigera*, which allows the direct comparison of these species. Our working hypothesis therefore is, that *E. timida* and *E. cornigera* will display significant differences for each of the factors examined here despite experiencing the same environmental conditions, which supports the idea that each species has physiological differences regarding functional kleptoplasty and starvation. To exclude the effects of irradiance – which varies highly in each natural environment, both species were placed under the same artificial lighting conditions. *E. cornigera* were also transitioned to *A. acetabulum* to avoid any effects of the algal species.

## Methods

### Field collection and lab conditions

Four aquaria containing lab-cultured *Acetabularia acetabulum* were transferred from an 18 °C culture room to a 22 °C room in March, 2016. This culture was established using specimens collected the previous year. The temperature was slowly increased using tank heaters until it reached 25 °C. Two of these tanks were gradually introduced to water with a lower salinity to match that of the Caribbean Sea: 36.5 psu seawater (from Mediterranean average 37.8–38.6 psu (NOAA). This was done to ensure that the algae consumed by *E. cornigera* did not suffer any effects of a sudden change in salinity.

Adult *Elysia cornigera* (*n* = 50) were collected on Spanish Harbor Key (approximately 24°64′97.83“N, 81°31′67.57”W) in April, 2016. They were removed from rocks covered in *Acetabularia crenulata* at a depth of 0.3–1 m. The surface water temperature was 25 °C. Animals were relocated to Bonn, Germany for experimentation. In Bonn, they were first acclimatized to artificial seawater and a 12 h L: 12 h D artificial light cycle (220 μmol quanta m^− 2^ s^− 1^) for a week. They were then transitioned to the food alga *A. acetabulum* (previously acclimatized to 25 °C) and allowed a further three acclimatization weeks to ensure most if not all of their sequestered chloroplasts derived from *A. acetabulum* rather than *A. crenulata*.

Adult *E. timida* (*n* = 40) were collected in July, 2016 in Blanes, Spain on rocks covered in *A. acetabulum* at 3–7 m depth*.* The water temperature was 23.3 °C when measured (measured at 7 m depth, surface temperature 24.2 °C). Animals were transported to Bonn, Germany and acclimatized to laboratory conditions for a week. Artificial lighting provided 220 μmol quanta m^− 2^ s^− 1^ light for 12 h L: 12 h D. Large tanks (50-90 L) containing 25 specimens and abundant food algae were then slowly warmed to 25 °C over the course of a week, by placing the tank in a 22 °C room and using tank heaters. They were then given a week to feed and acclimatize to the higher temperature before experimentation began. To better reflect the natural acclimatization to a broader range of temperatures, data already available from specimens in the same wild population (Blanes, Spain) are included here [37]. These animals were collected during fall and spring seasons and kept according to their actual and natural acclimatization at 18 °C and 21 °C respectively, in the same room, same tanks, under the same light regimes as the 25 °C *E. timida* (summer population) and *E. cornigera* populations.

**Fig. 1:**
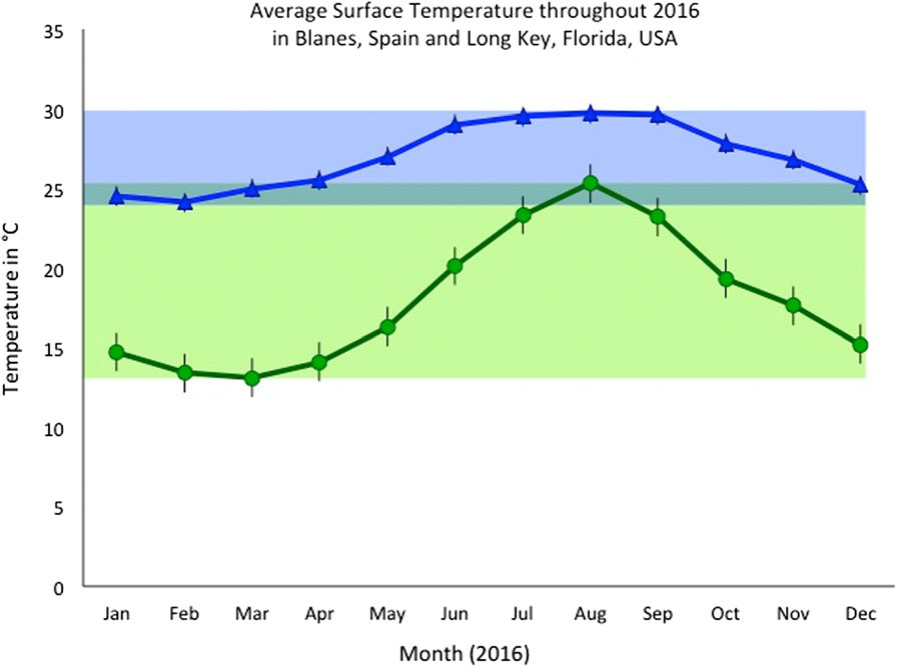
The average sea surface temperature in Blanes, Spain and Spanish Harbor Key, Florida, USA for 2016 according to satellite maps provided by NOAA. The monthly average for Blanes, Spain is depicted with a green line and the range is shaded in green showing *E. timida’s* natural temperature range. *E. cornigera’s* natural temperature range is shown by blue shading and the blue line depicts the monthly average temperature. The overlap in these ranges occurs at 25 °C and is depicted by bluish-green shading

**Fig. 2:**
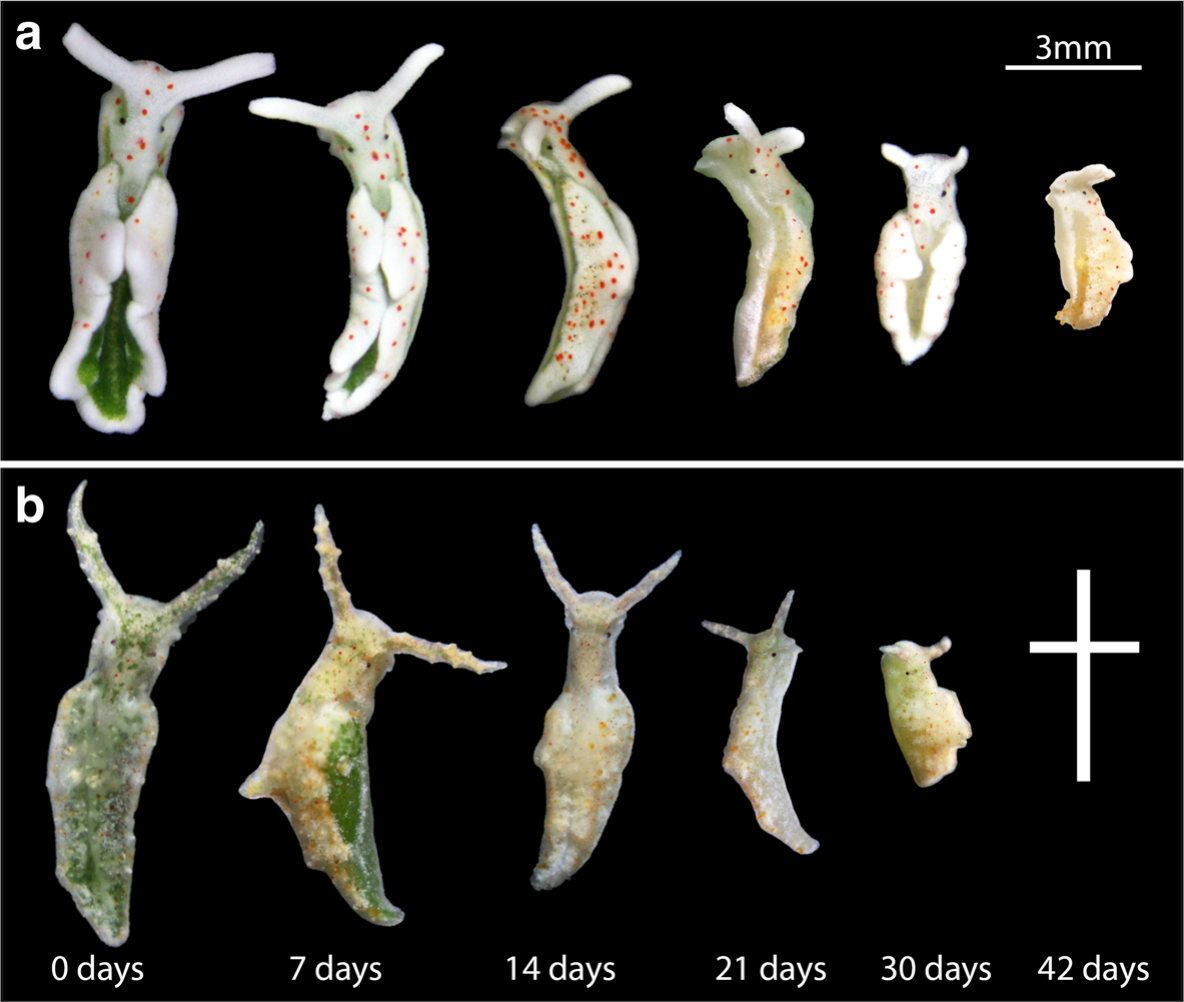
Species investigated during starvation. **a** *E. timida* during starvation at 25 °C. The loss of green pigmentation is due to the loss of chlorophyll a within the slug. **b** *E. cornigera* during starvation. The cross (†) at the end of the *E. cornigera* series indicates that this species did not survive starvation up to 42 days

*E. cornigera* were not subjected to multiple temperature treatments for multiple reasons including such as: our investigation set out to examine these species under natural conditions and *E. cornigera* has not been reported from water colder than ~ 24 °C (Fig. 1; 2) subjecting *E. cornigera* to temperatures lower than those which they are naturally adapted could have a variety of different effects and would not reflect the natural conditions in which they are found and to which they are adapted; 3) collection limits necessitated prioritizing a limited number of animals for each experiment and prohibited studying artificially induced colder temperatures.

### Laboratory procedures

The sample time points chosen for *E. timida* 25 °C were 0, 7, 14, 21, 30, 42 days in starvation, to match the time points from the fall and spring season surveyed in Laetz et al. [37]. Sixty and 89 days were planned but the slugs did not survive past 42 days. *E. cornigera* were sampled at 0, 7, 14, 21 days. Ten specimens of each species were placed in 25 °C aquaria and each specimen’s starvation longevity was recorded. For both *E. timida* and *E. cornigera,* three Pulse Amplitude Modulated Fluorometry (PAM) measurements were taken for nine specimens, after 15 min of dark acclimatization, for each time point, according to the protocol established by Wägele and Johnsen [18]. Pulse Amplitude Modulated fluorometry (PAM) is a technique that allows the calculation of values, which reflect the overall photosynthetic efficiency of chloroplasts within a tissue (maximum quantum yield of photosystem II). These values are expressed as a percentage (the relative value F_V_/F_M_).

For the following experiments, three specimens from each species were used per time point and per temperature treatment although extra individuals were also included in case they were needed (*E. timida* used in experiment: *n* = 24; *E. cornigera* used in experiments: *n* = 12). They were photographed (Fig. 2) and stained by placing each specimen in a 5 μmol acridine orange and filtered seawater solution for 30 min [37]. They were then vivisected and placed on a microscope slide for confocal microscopy on a Leica SPE Confocal Laser Scanning Microscope (CLSM). Five different cross-sections were scanned to gather an impression of the digestive activity throughout the entire animal. Each scan was 8 μm deep, encompassing the entire thickness of the digestive gland tubule wall cells, while avoiding digestive gland lumen and tissue outside the digestive gland. Acridine Orange is excited by the blue laser (488 nm) and emits photons at 645–670 nm (dimer type optimum 656 nm) in extremely acidic environments such as lysosomes. Functional chloroplast abundance was also measured during these scans, by capturing chlorophyll a autofluorescence with blue laser excitation (488 nm) and 600-640 nm as the accepted emission range (chlorophyll optimum 633 nm) [37].

### Excrement surveys

Throughout the experimental processes, *E. timida* and *E. cornigera* were kept in tanks of 25 animals, without access to food. Weekly cleaning inhibited the growth of algae in these tanks. Excrement samples from each tank were examined weekly to determine if chloroplasts were present, indicating the decreases in functional chloroplast abundance is due to excretion rather than digestion. This was accomplished using the CLSM to look for chlorophyll a autofluorescence in intact chloroplasts for each of the excrement samples.

### Statistical analyses

PAM values (*n* = 9) and starvation longevities (*n* = 10) were compared using QQ plots to assess normality and populations best modeled by linear functions with an r^2^ value > 0.90 were considered normally distributed. Levene’s test was applied to assess homogeneity of variance once normality was established. When the assumptions for a one-way standard ANOVA were met, this test was used to ascertain if differences in the recorded values were significant for each time point. Tukey HSD post hoc testing was used to compare amongst the individual treatments when the ANOVA proved significant. For treatments where standard ANOVA assumptions were not met (heterogeneous variances), a Welch ANOVA and subsequent post hoc Games-Howell tests were used instead. The result form each analysis can be found in Additional file 1. Functional chloroplast abundance, lysosome abundance and excrement samples were not statistically analyzed since the number of replicates was too low (*n* = 3).

## Results

### Starvation times

Three *E. timida* temperature conditions are compared here (*n* = 10/treatment), to determine if any differences in survival rate are likely due to temperature. *Elysia timida* starved at 25 °C survived for a mean 42.4 ± 3.37 days, *E. timida* 21 °C withstood 43.3 ± 2.91 days, *E. timida* 18 °C survived 95.90 ± 3.84 days, while *E. cornigera* survived a mean 29.4 ± 1.78 days. Each population was assessed with a QQ Plot revealing a strong indication of normally distributed data (as indicated by a linear best-fit model and an r^2^ value > 0.90). Levene’s testing for homogeneity of variance was not significant (*p* = 0.23) suggesting homogeneous variances across each group. One-way ANOVA and Tukey HSD analyses reveal a significant difference in the duration starving *E. timida* (all populations *p* < 0.01) can withstand as compared here to *E. cornigera.* Within the *E. timida* temperature treatments, *E. timida* 18 °C survived significantly longer than both *E. timida* 21 °C (*p* < 0.01) and *E. timida* 25 °C (*p* < 0.01) although *E. timida* 21 °C and *E. timida* 25 °C did not display significantly differing starvation longevities (*p* = 0.53). The results from each statistical test can be seen in Additional file 1.

### Photosynthetic efficiency

QQ plots for each treatment at each point during starvation allowed the assumption of normally distributed data except for *E. cornigera* starved for 30 days*.* This non-normality is likely due the high frequency of zeros found in the dataset and a lack of equipment sensitivity for very low values. Levene’s test for each time point displayed heterogeneity of variance after the control, 0 days in starvation time point. Therefore, Welch ANOVAs were conducted for all of the subsequent comparisons.

**Fig. 3:**
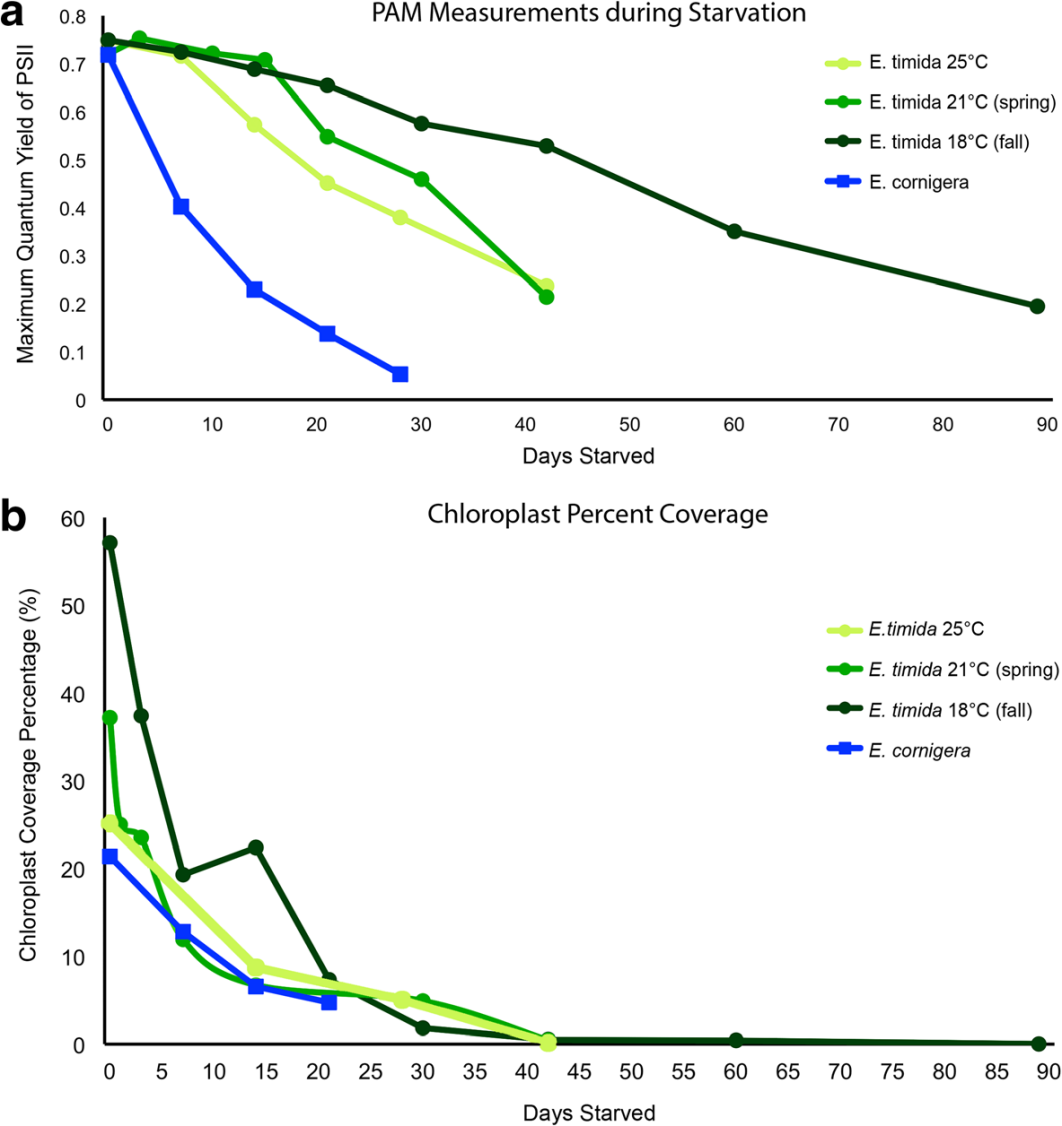
Functional chloroplast efficiency and abundance. **a** The chloroplast efficiencies (PAM values) of *E. timida* in each temperature treatment and *E. cornigera. E. cornigera* drops in photosynthetic efficiency more rapidly than any of the *E. timida* treatments. Each point is the average of three independent measurements following dark acclimation. *E. timida* at 25 °C is denoted by light green circles, at 21 °C by medium green circles and at 18 °C by dark green circles. *E. cornigera* at 25 °C is represented by blue squares. Lines connecting each point display the average rate of decrease. **b** Percent chlorophyll coverage throughout starvation for each examined species. This was computed by measuring the area (in each image, for each stack) that was covered in Digestive Gland Tubule (DGT) and the remaining area – designated as Non-Digestive Gland Tissue. The area covered by functional chloroplasts inside the DGT was divided by the total DGT producing a relative value, a percent of the DGT that is filled with chloroplasts. This value was averaged for each measurement taken (8 images/stack, 5 stacks/specimen, 3 specimens/time point totaling 120 measurements for species at each time point) and graphed here to show the overall trend for each species at each time point. *E. timida* 18 °C is shown by dark green circles, 21 °C by medium green circles and 25 °C by light green circles. *E. cornigera* is depicted by blue squares. Error bars are not depicted here for graph clarity and can be found in the text

Each *E. timida* temperature treatment, 18 °C, 21 °C and 25 °C, and *E. cornigera* begin their starvation periods with high PAM values (F_V_F_M_), having averages of 0.749, 0.73, 0.765, and 0.728 respectively. No significant difference was recorded amongst these treatments (*p* = 0.08). After only 7 days however, the *E. timida* populations group significantly with high PAM values: 0.723 (18 °C), 0.721 (21 °C) and 0.760 (25 °C) (*p* < 0.17), while *E. cornigera* diverges, dropping rapidly to a mean 0.401 (*p* < 0.01). While each of the *E. timida* treatments and *E. cornigera* decrease, the rate of PAM value decrease differs. Welch ANOVAs reveal that after the 0-day time point, *E. cornigera* PAM values are always significantly lower than each of the *E. timida* treatments. PAM averages throughout their starvation periods for each of the species examined are summarized in Fig. 3a and the results from each statistical test can be seen in the Additional file 1.

### Functional kleptoplast abundance

**Fig. 4:**
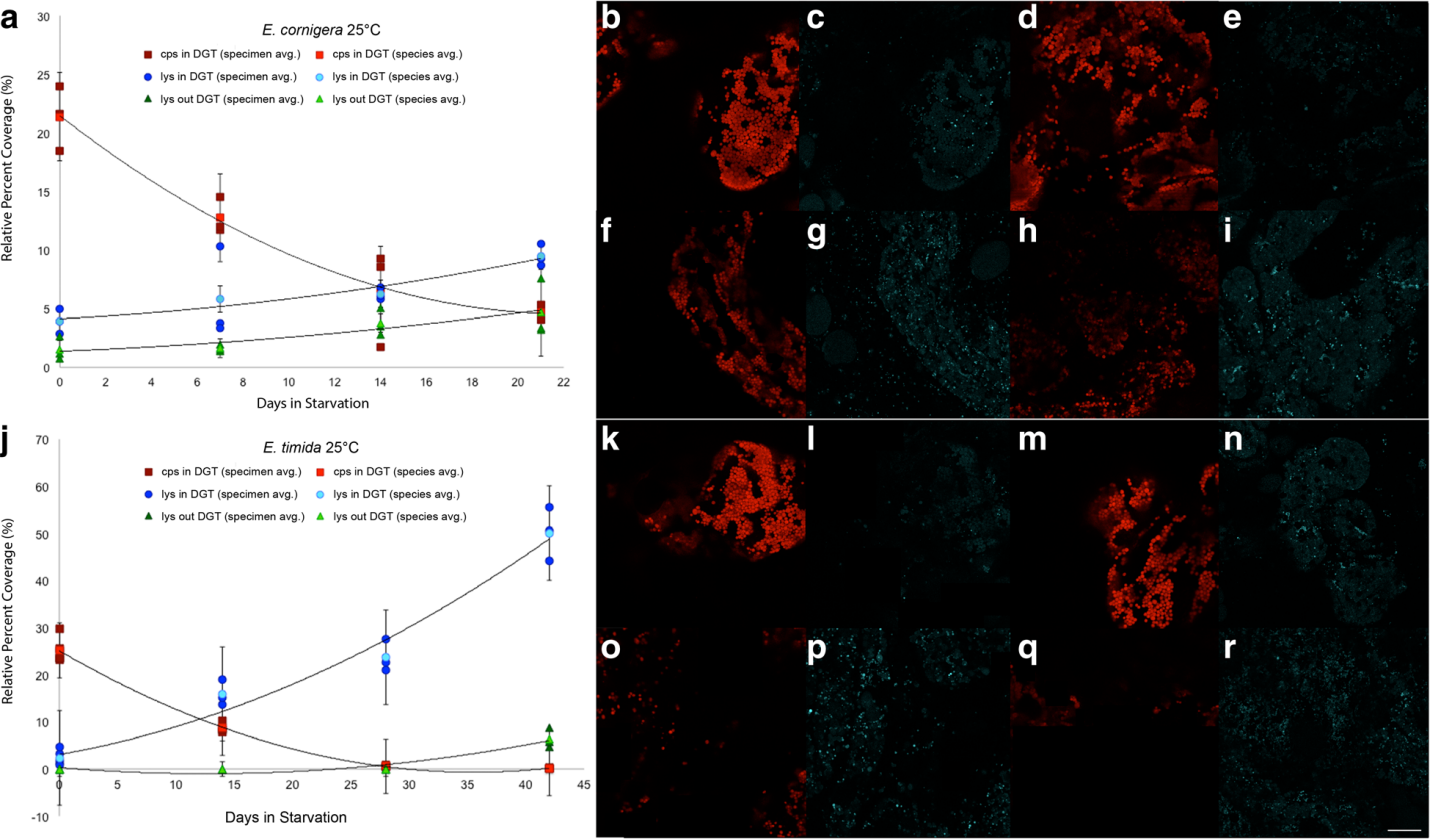
Functional chloroplast (cps) and lysosome abundance (lys) in *E. cornigera* and *E. timida* 25 °C. **a**-**i** *E. cornigera.* **j**-**r** *E. timida.* Functional chloroplasts are depicted by red squares, the dark red ones showing the average per specimen (*n* = 3) and the light red squares showing the average of these three specimens representing the species average at each time point. Lysosomes within the digestive gland tubules (DGT) are illustrated with blue circles, the dark circles again showing the specimen average and the light circles revealing the species average. Lysosomes outside the DGT are demonstrated by green triangles, the dark depicting the specimen average and the light green indicating the species averages. For clarity, error bars (standard error) are only shown for the species average. **b** *E. cornigera* cp abundance at 0-days starved. Cps are falsely colored red. **c** *E. cornigera* lys abundance at 0-days starved. Lys are falsely colored blue. **d**, **e** *E. cornigera* – 7-days starved. **f**, **g** *E. cornigera* – 14-days starved. **h**, **i** *E. cornigera* – 21-days starved. **j** Cp and lys abundance in *E. timida* starved at 25 °C. Functional cps and lys in/out DGT are depicted as described above for (**a**). **k** Cps in *E. timida* 25 °C starved for 0 days. **l** Lys in *E. timida* 25 °C starved for 0 days. **m**, **n** *E. timida* 25 °C – 14 days starved. O, **p** *E. timida* 25 °C – 21 days starved. **q**, **r** *E. timida* 25 °C – 42 days starved. Error bars are not depicted here for graph clarity and can be found in the text. Scale bar – 50 μm

Kleptoplast abundance was also recorded for each time point during the starvation period for each *E. timida* temperature treatment and *E. cornigera.* While both species decline in the number of functional chloroplasts, the number of incorporated plastids at the beginning of the starvation period and rates at which they decrease differ (Fig. 3b). *E. cornigera* has the lowest functional chloroplast retention time and some specimens died with some functional kleptoplasts in their bodies after 21 days (Fig. 4a, h). Both 18 °C and 21 °C *E. timida* populations contained functional chloroplasts until 42 days, while the warmer *E. timida* (25 °C) lost almost all functional chloroplasts earlier, after only 30 days (Fig. 4j, q). An overview of chloroplast decrease in 25 °C *E. timida* and *E. cornigera* can be seen in Fig. 4. Minuscule amounts of chlorophyll a were found in *E. timida* 18 °C until 60 days, however the amounts measured are so low they are likely remnants found in degraded thylakoids.

### Lysosomal activity

**Fig. 5:**
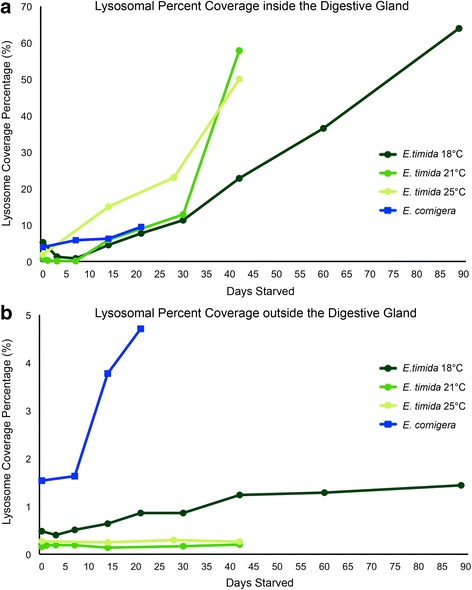
Lysosome abundance examined throughout a starvation period. **a** Percent Lysosome Coverage inside the Digestive Gland Tubule (DGT). **b** Percent Lysosome Coverage outside the DGT. The data in (**a** and **b)** were computed following the same procedure as described in Fig. 3 for functional chloroplasts. *E. timida* 18 °C is shown by dark green circles, 21 °C by medium green circles and 25 °C by light green circles. *E. cornigera* is depicted by blue squares. Error bars are not depicted here for graph clarity and the values can be found in the text

Lysosomal activity inside the digestive gland tubules is presumably involved in digestion, and used here as an indication of digestive activity in these animals. Each of the *E. timida* populations surveyed here followed the same trend, a very low lysosomal abundance at the beginning of the starvation period (0–5 ± 0.22% of the digestive gland total area covered by lysosomes) and a large increase (48–60 ± 3.4% total lysosome coverage) in the second half of the starvation period (differing time spans depending on the temperature acclimatized population) (Fig. 4j and 5a). The averages presented here are the mean lysosome abundance values based on three specimens per time point (15 measurements/specimen). These populations differ however, in the rate of lysosomal increase. 25 °C *E. timida* have an almost linear increase, modeled best by the function: y = 1.16× - 0.51 (r^2^ = 0.99). The 18 °C and 21 °C *E. timida* relative abundances do not increase linearly, instead modeled best by the quadratic functions: y = 0.0054 × ^2^ + 0.23× + 0.99 (r^2^ = 0.99) and y = 0.052 × ^2−^ 1.07× + 3.69 (r^2^ = 0.95). The *E. cornigera* specimens examined display a slight increase in lysosome coverage, from 3.9–9.4 ± 1.1% over the 21 days they starved (Fig. 5a).

While lysosomes are an integral part of normal cellular machinery, activity increases above the baseline normal background level, outside the digestive gland, may be indicative of autophagy. Only *E. cornigera* had changes in the relative percent coverage of lysosomes outside the digestive gland*. E. cornigera* began the starvation period with a 1.5 ± 0.31% non-digestive gland tubule percent coverage, and increased to 4.7 ± 1.1% during the 21-day starvation period. The different *E. timida* populations displayed minor fluctuations in their percent coverage (0–0.5 ± 0.02%), but never increased to even 1% percent throughout the entire starvation period (Fig. 5b).

### Excrement

**Fig. 6:**
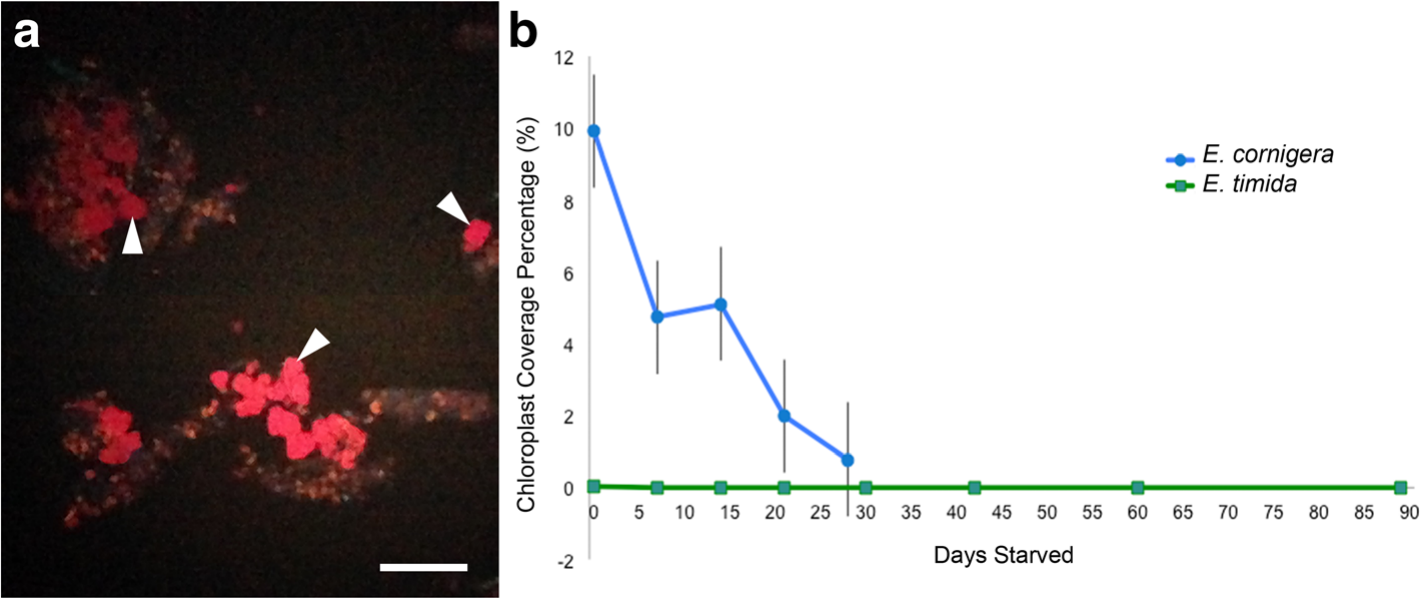
Functional chloroplasts in *E. timida* and *E. cornigera* excrement. **a** *E. cornigera* excrement containing a high density of functional kleptoplasts (red circles, a few indicated by arrowheads). **b** Functional chloroplast density within *E. timida* and *E. cornigera* excrement. Since *E. timida* at each temperature treatment lacked chloroplasts in their excrement, they were all graphed as a single *E. timida* line (green squares). The excreted plastid abundance in *E. cornigera* is depicted by blue circles

Excrement samples were examined to determine whether or not functional chloroplasts were being excreted throughout the starvation period. Excreted chloroplasts were discovered in *E. cornigera* samples throughout the starvation period although chloroplasts were never observed in samples from any of the *E. timida* populations (Fig. 6a, b). Excreted plastids were still round in shape, appearing intact. Nothing about the chloroplasts found in the *E. cornigera* excrement appeared different from those found within the slug, except for the debris surrounding them (Fig. 6a).

## Discussion

The duration each *E. timida* treatment from this shallow sea population could survive depends on the temperature conditions to which it was acclimatized. Both *E. timida* treatments that experienced elevated temperatures (21 °C and 25 °C), lived for a significantly shorter time span, only reaching 42–50 days, while *E. timida* at 18 °C, the temperature recorded in their natural habitat during the fall, survived over 100 days (maximum from this study: 102 days). Interestingly, *E. timida* at 21 °C do not survive longer than those at 25 °C. Despite this, temperature may still explain the reported discrepancies for *E. timida* longevity in starvation, chloroplast abundance, starch abundance and photosynthetic efficiency [37, 49]. *E. cornigera* only survived a maximum of 32 days in the laboratory, suggesting that elevated temperatures shorten *E. timida’s* life expectancy, but not to the same degree that *E. cornigera* naturally experiences. This indicates that lower temperatures may facilitate *E. timida’s* long-term survival but temperature is not the only factor distinguishing these species from one another regarding longevity in starvation.

The photosynthetic efficiencies of *E. cornigera* and *E. timida* have been reported in previous investigations, and while water temperatures were often reported, these experiments were conducted under a variety of different conditions (different field sites, lab setups, collection times) so comparison to our study risks unintended error [6, 8, 17, 50]. In this study, PAM values are recorded for *E. timida* acclimatized naturally according to environmental temperatures and then accordingly cultivated at 18 °C, 21 °C and 25 °C and *E. cornigera* at 25 °C under standardized conditions. Each *E. timida* temperature group and the *E. cornigera* began with a statistically similar value but diverged rapidly, suggesting both *E. timida* and *E. cornigera* that are fed the same food algae and kept under the same conditions will sequester chloroplasts with the same photosynthetic efficiencies; however once starvation begins, the photosynthetic efficiencies change depending on the temperature and species. Since algae from the same culture was provided each species and the algae for 25 °C *E. timida* and *E. cornigera* was also acclimatized to 25 °C, the differing declines in photosynthetic efficiency between 25 °C *E. timida* and *E. cornigera* indicate a difference in the way chloroplasts function and survive in the digestive gland cells of these two species. This suggests the slug species itself is at least partially responsible for the health and longevity of the chloroplasts within its digestive gland and the ability of a chloroplast to be retained is not solely due the algal species involved.

The declines in *E. timida* photosynthetic efficiency presented in this study differ from the photosynthetic efficiencies measured by Schmitt et al. [8], which also examined *E. timida* under different temperature regimes. In that study, the temperatures were not closely controlled (fluctuating between ~ 19–24 °C depending on the season), and this may explain why their populations did not reveal distinct grouping based on the temperature as they did in this study. Despite the lack of a standardized temperature, both of these studies confirm that higher temperatures lead to a faster decline in photosynthetic efficiency during starvation.

Functional kleptoplast abundance within the digestive gland tissue of multiple species has now been analyzed [37, 51–53]. This analysis adds *E. cornigera* and *E. timida* starved at 25 °C to determine if there are differences in chloroplast metabolism inherent to the slugs’ cells that are independent to the natural temperatures these animals encounter and the results here indicate this to be the case. Each of the treatments investigated here reveal a different rate of functional chloroplast decrease, with no two treatments sharing a similar rate, although they all decrease. Despite consuming the same algal species and living in side-by-side aquaria under the same lab conditions (light, temperature…), *E. timida* 25 °C and *E. cornigera* do not display the same decrease in functional chloroplasts. *E. cornigera* were even observed dying with functional chloroplasts inside their digestive gland tubules, while none of the *E. timida* temperature treatments were observed containing functional plastids at their time of death. The only discernable abiotic factor that was different in each tank was the salinity of the water, which was always matched to the natural conditions each species encounters in the field. The effects of salinity on functional kleptoplasty require further investigation to determine whether or not it influences chloroplast vitality.

Two other kleptoplast retaining species, *Thuridilla hopei* (StR) and *Elysia viridis* (StR or LtR depending on the ingested algae) have been investigated regarding digestive activity, kleptoplast abundance and the potential autophagal response [37]. Despite confirmation that both species are StR forms, *T. hopei* and *E. cornigera* do not display the same pattern regarding functional chloroplast abundance in their tissues. They survive about the same time in starvation, however *T. hopei* is devoid of functional chloroplasts after only 14 days (confirming observations made by Martin et al. [54]) whereas *E. cornigera* dies with some functional chloroplasts still incorporated. *E. viridis,* which is sometimes considered a StR species also differs in functional chloroplast abundance, having no sign of functional chloroplasts by the end of its starvation period, but lasting an average of 10 days longer in starvation. *E. viridis* is enigmatic however, having various starvation longevities that may depend on the algae ingested, and exhibiting various photosynthetic efficiencies in starvation, so further research is required to facilitate a proper comparison to this species [35, 40, 54].

Two of the *E. timida* populations (21 °C and 25 °C) showed a faster decline in the number of functional chloroplasts, lower number of sequestered plastids at the beginning of the starvation period and faster death by starvation, however they still survived starvation longer than *E. cornigera* (this study), *E. viridis* and *T. hopei* [37]. A comparison of all of the *E. timida* populations here also shows that temperature does not seem to affect the rate of functional chloroplast decrease in the tissues, since 25 °C *E. timida* have a higher rate of decrease than the 21 °C and 18 °C groups. While 18 °C *E. timida* lived the longest, their functional plastid abundance was almost non-existent after 42 days, the same time span observed in 21 °C *E. timida* and 25 °C *E. timida.* These results seem to contradict results that show the maximum amylose concentration inside kleptoplasts occurring at 42 days in 18 °C starved *E. timida,* which also indicates the presence of these plastids since amylose is accumulated within the plastid [49]. However, this discrepancy is likely due to the means by which kleptoplast abundance/presence was assessed. Upon examining histological sections, it is clear that kleptoplasts are still present until after 63 days [49], however if they do not contain structurally intact chlorophyll a, they will not be detected using chlorophyll a autofluorescence. This highlights the need for a method that accurately quantifies kleptoplast abundance in these slugs and does not rely on autofluorescence.

Lysosomal activity within the digestive gland tubule network was monitored as an indicator of digestive activity in these tissues. Two different patterns were observed amongst the species examined. Species with low longevities in starvation, *E. cornigera, E. viridis* and *T. hopei,* display a steady increase in lysosomal abundance during their starvation periods [37]. *E. timida*, however, can withstand a much longer duration without food and exhibits a two part pattern: the first half of the starvation period is marked by a slight increase in the number of lysosomes while the second half of the starvation period reveals a faster rate and significantly higher lysosome abundance. This pattern occurs within each of the temperature treatments investigated here suggesting temperature and lysosome production are not correlated in this species.

Lysosomal activity outside the digestive system is not involved in an organisms digesting foreign material as food, but rather comprises an organism’s internal digestion of its own cellular components as part of normal cellular functioning and as autophagy. There was no observable difference between *E. timida* at 18 °C and 21 °C when compared to *E. timida* 25 °C and these populations closely resembled those observed for *E. viridis* [37]. Neither *E. cornigera* nor *T. hopei* displayed a trend that aligned with *E. timida* or *E. viridis,* indicating each of these species has a different autophagal responses during starvation. The strong increase in lysosomes produced by *E. cornigera* and *T. hopei* after 7 days in starvation suggests an activation of autophagal procedures in these animals. This furthers conclusions made by de Vries et al. [45] when they examined metabolic transcription in *E. timida* and *E. cornigera,* and revealed elevated expression rates of autophagal genes in *E. cornigera* after 7 days in starvation, which were not present in *E. timida.* Interestingly, *E. timida* and *E. viridis* do not exhibit a visible increase, suggesting the autophagal processes behind their decreased size are not detected using the acridine orange method. Fully understanding the autophagal processes in these species will require further investigations.

The excrement surveys conducted here reveal that *E. cornigera* do not digest all of the chloroplasts they sequester. The chloroplast abundance within the excrement samples deceases throughout the starvation period and since no new chloroplasts were introduced to these animals, it is clear that some chloroplasts were retained for weeks within the digestive gland before finally being excreted. The cause for this delay is unknown, and requires further investigation, but may be due to Reactive Oxygen Species (ROS) buildup around these chloroplasts. ROS accumulation was examined by de Vries et al. [45] in *E. cornigera* and *E. timida*, the former showing increases in both hydrogen peroxide (H_2_O_2_) and superoxide (O_2_^−^) during starvation. Plastid excretion and autophagy (as seen with the lysosomes in- and outside the digestive gland) by *E. cornigera* may be a response to the buildup of these cytotoxic molecules. Each of the *E. timida* temperature groups lacked plastids in their excrement, showing that *E. timida* do digest the chloroplasts they sequester. *E. timida* also lack an increase in ROS during starvation [45], although this was only monitored until the 30-day starvation point, which likely explains the first half of the *E. timida* starvation period, where lysosomal activity is low. Further investigations are needed to confirm if ROS levels increase in *E. timida* after 30 days, correlating to the lysosomal activity increases observed here. This fundamental difference in the way *E. timida* and *E. cornigera* react to their sequestered plastids may be exactly what allows *E. timida* to survive 3–4 times as long as *E. cornigera* in starvation and ROS could be implicated in the digestive activity differences observed here.

**Fig. 7:**
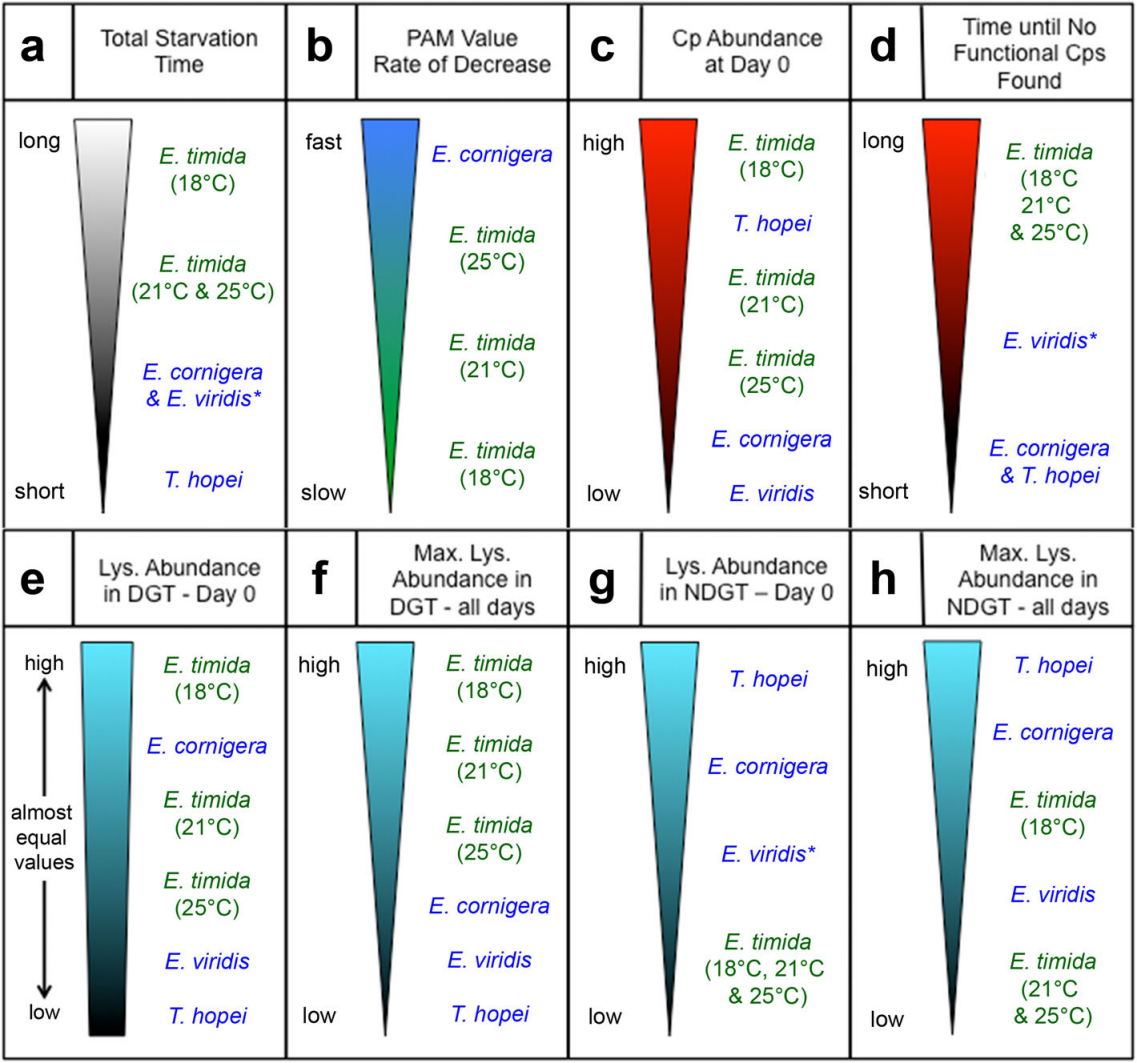
Overview of the digestive trends in the examined species. To visualize the overall trends observed in each of these populations, for each experiment, the numbers were removed and each population was ranked. The colored triangles represent the quantity of each factor (for example: number of chloroplasts in unstarved animals) from large to small. Each population is listed according to rank. Species written in green are the traditional long-term chloroplast retaining species whereas those in blue are the short-term retaining species. *E. viridis* is designated in blue with an asterisk because it more closely resembles an StR form in this investigation, but has been classified as LtR in the past. **a** Total longevity in starvation from a long duration to short. *E. timida* 18 °C has the longest longevity in starvation and *T. hopei* has the shortest. The triangle is colored black and white to represent alive/dead. **b** PAM value rate of decrease. *E. cornigera* have the fastest decrease in photosynthetic efficiency during starvation. The triangle is blue/green because StR is often depicted in blue and LtR in green. **c** Functional chloroplast abundance in unstarved (Day 0) Slugs. Throughout this investigation, chloroplasts are indicated in red because of their autofluorescence. **d** The number of days until functional chloroplasts are no longer observed in the digestive gland. **e** Lysosome abundance within the Digestive Gland Tubule (DGT) for unstarved specimens (Day 0). A quadrilateral rather than triangle is drawn because the values are very similar. It is colored blue aligning with the pictures of lysosomes presented in this investigation. **f** The maximum lysosome abundance throughout the entire starvation period within the DGT. These maximums occur at different time points for each species. **g** Lysosome abundance in Non-Digestive Gland Tissue (NDGT) at Day 0. **h** Maximum lysosome abundance in NDGT throughout the starvation period

The overall differences observed in each of the species examined here suggest the complexity behind functional kleptoplasty. *E. cornigera, T. hopei* and *E. viridis* have all been previously labeled StR forms, which is based on photosynthetic efficiency [17]. Despite this, when another factor is examined, such as functional kleptoplast abundance, this category no longer appropriately groups these organisms. When each of the species examined here is reduced to a trend and compared to the other’s trends, it is clear how the LtR, and StR category labels fail to group these organisms in consistent groups. Figure 7 (specifically C, E and H) compares each species with each of the factors investigated here, to show how this grouping proves inadequate when examining something other than photosynthetic efficiency amongst these animals. Based on this, we therefore suggest that these labels only be used when referring to photosynthetic efficiency amongst sacoglossan slugs. For other factors that have been or may be investigated, such as functional plastid abundance and lysosomal activity, the lack of natural clustering should be kept in mind and these categorical labels avoided.

## Conclusions

Put simply, *E. cornigera* is not warmed up *E. timida* incapable of surviving long starvation periods due to higher temperatures. Physiologically, these two species are not the same when it comes to responding to starvation, even under the same temperature conditions, and these dissimilarities require further investigation. Temperature did affect *E. timida’s* photosynthetic efficiency and total starvation time, but could not be correlated to increased digestive rates, autophagy or the decline of functional chloroplasts in these tissues. When the comparison is broadened to include all of the species examined, it is clear that each species reacts to starvation differently, some excreting their chloroplasts – others digesting, some showing signs of autophagy – others not, which defies our ability to assume similarity between sacoglossan species and reveals the complex nature of functional kleptoplasty in these animals.

## Additional file

Additional file 1: Statistical overview. (PDF 3530 kb)

## Abbreviations

AO: Acridine orange
CLSM: Confocal scanning laser microscope
LtR: Long-term kleptoplast retaining species
PAM: Pulse amplitude modulated fluorometer
PSU: Practical salinity units
ROS: Reactive oxygen species
StR: Short-term kleptoplast retaining species
