# Supplementary material for: How does temperature affect functional kleptoplasty? Comparing populations of the solar-powered sister-species Elysia timida Risso, 1818 and Elysia cornigera Nuttall, 1989 (Gastropoda: Sacoglossa)
Source: Front Zool. 2018 Apr 24;15:17. doi: 10.1186/s12983-018-0264-y (PMC5937827; doi:10.1186/s12983-018-0264-y)
Supplement: Supplementary file 1 — Statistical overview. (PDF 3530 kb) [file 12983_2018_264_MOESM1_ESM.pdf]

Supplemental File 1 - Statistical overview

Section 1: Starvation Longevity

Normality testing – QQ plots

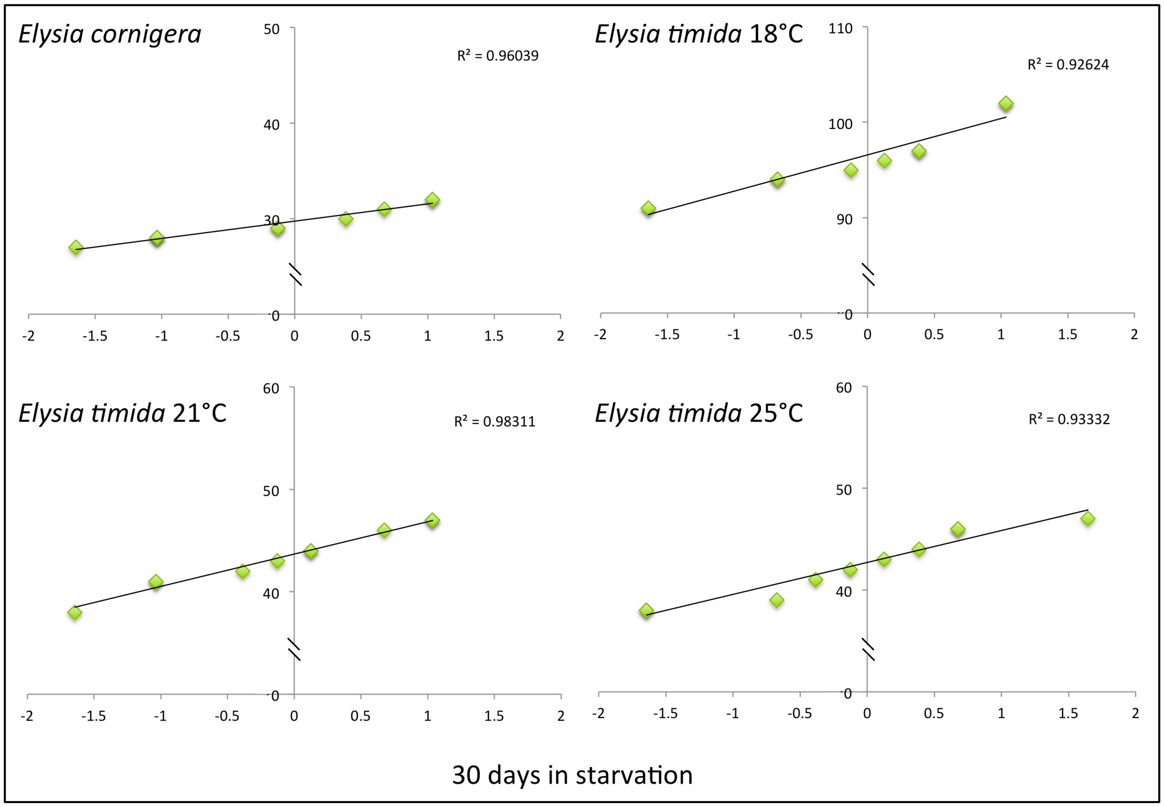

Interpretation: The populations are assumed to have a normal distribution since the data points are best fit by straight lines and display r^2 values > 0.90.

Starvation Longevity - Levene's Test for Homogeneity of Variance:

Levene ANOVA –Starvation Longevity				
	***E. timida* 18°C**	***E. timida* 21°C**	***E. timida* 25°C**	**E. cornigera 25°C**
**n**	10	10	10	10
**Sum**	29	23	28	14.8
**Mean**	2.9	2.3	2.8	1.48
**Sum of squares**	132.9	76.1	102.4	28.4
**Variance**	5.4222	2.5778	2.6667	0.7218
**Standard dev.**	2.3286	1.6055	1.633	0.8496
**Standard error**	0.7364	0.5077	0.5164	0.2687
**Summary**		**Degrees of**		
	**Sum of Squares**	**Freedom**	**Mean Squares**	**F statistic: 1.48**
**Treatment**	12.628	3	4.2093	**P value: 0.2363**
**Error**	102.496	36	2.8471	**not significant**

Interpretation: Examining the starvation longevity for each population reveals that the variance for each population can be assumed homogenous due to the insignificant p value failing to reject the null hypothesis. Therefore, a standard ANOVA analysis can be performed to compare these populations. Tukey HSD tests were used following the ANOVA as a post hoc analysis to compare between each group.

Standard ANOVA – Starvation Longevity				
	***E. timida* 18°C**	***E. timida* 21°C**	***E. timida* 25°C**	**E. cornigera 25°C**
**n**	10	10	10	10
**Sum**	959	433	424	294
**Mean**	95.9	43.3	42.4	29.4
**Sum of squares**	92101	18825	18080	8672
**Variance**	14.7667	8.4556	11.3778	3.1556
**Standard dev.**	3.8427	2.9078	3.3731	1.7764
**Standard error**	1.2152	0.9195	1.0667	0.5617
**Summary**		**Degrees of**		
	**Sum of Squares**	**Freedom**	**Mean Squares**	**F statistic: 919.45**
**Treatment**	26035.7	3	8678.5667	**P value < 0.0001**
**Error**	339.8	36	9.4389	**significant**

Tukey HSD results – starvation longevity				
E. timida 18°C	vs.	E. timida 21°C	significant	p < 0.01
E. timida 18°C	vs.	E. timida 25°C	significant	p < 0.01
E. timida 18°C	vs.	E. cornigera 25°C	significant	p < 0.01
E. timida 21°C	vs.	E. timida 25°C	not-significant	
E. timida 21°C	vs.	E. cornigera 25°C	significant	p < 0.01
E. timida 25°C	vs.	E. cornigera 25°C	significant	p < 0.01

<u>**Section 2: Pulse Amplitude Modulated Fluorometry (PAM)**</u>

Normality testing: QQ Plots

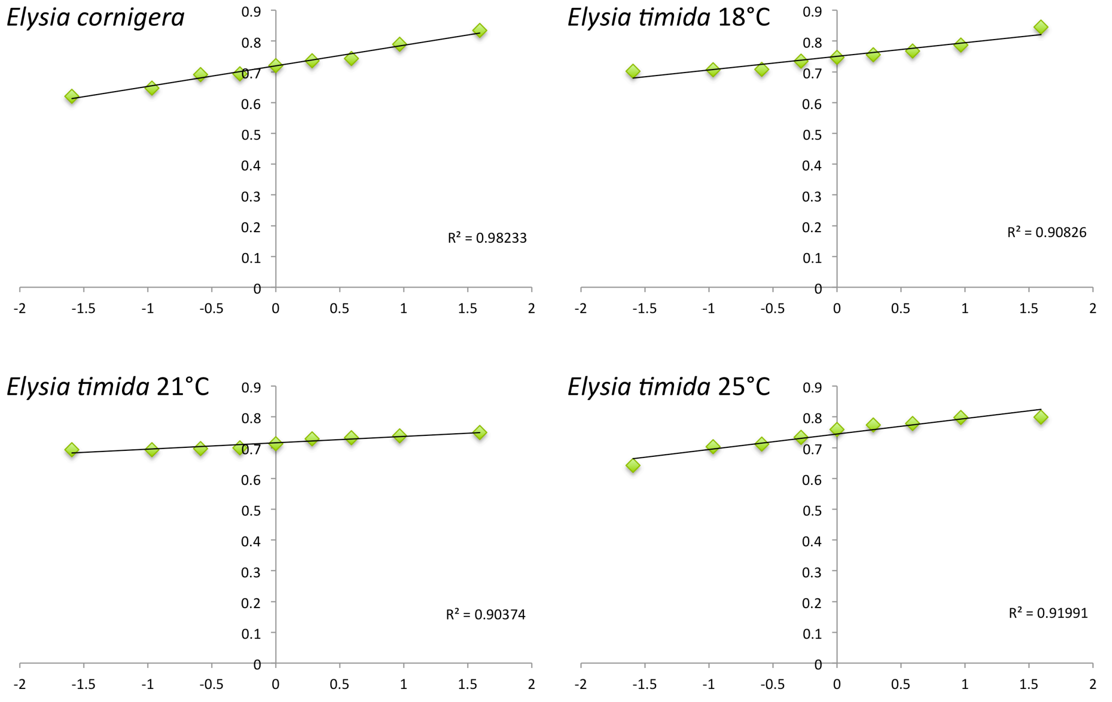

0 days in starvation (unstarved)

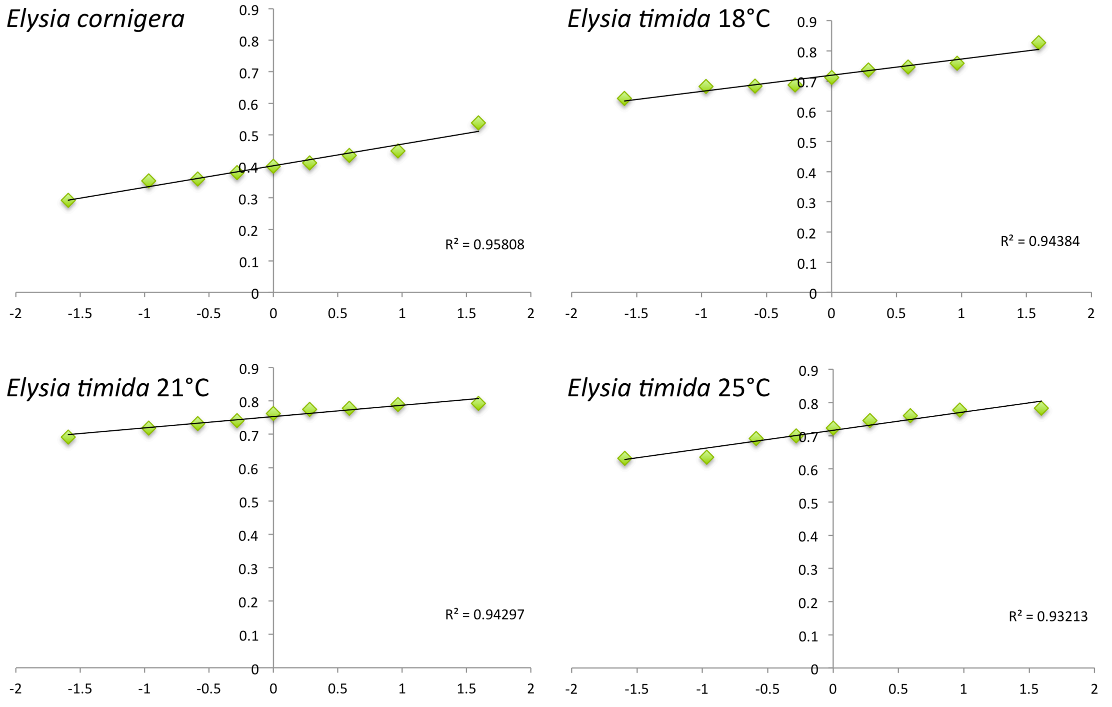

7 days in starvation

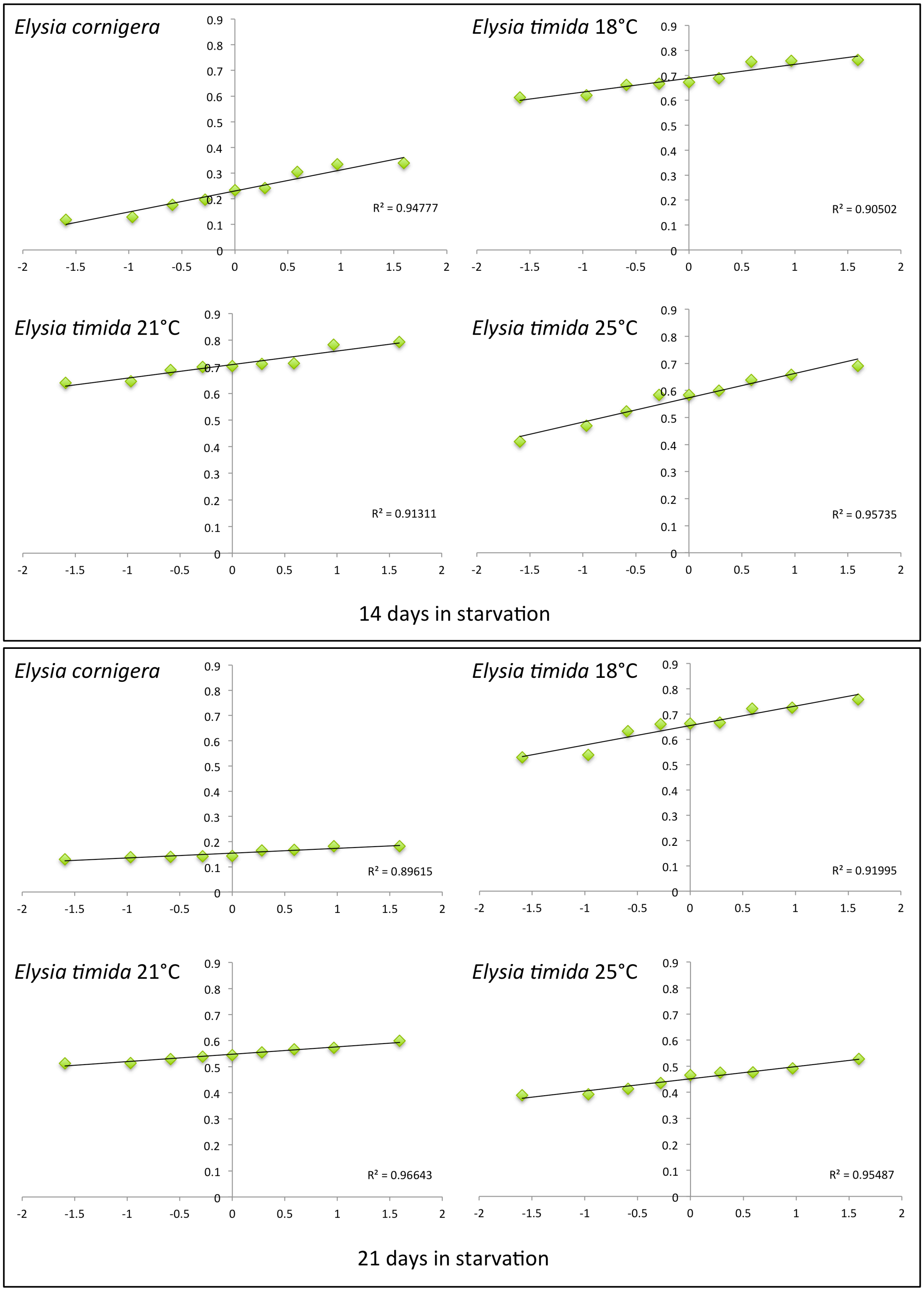
Elysia cornigera
R² = 0.94777
Elysia timida 18°C
R² = 0.90502
Elysia timida 21°C
R² = 0.91311
Elysia timida 25°C
R² = 0.95735
14 days in starvation
Elysia cornigera
R² = 0.89615
Elysia timida 18°C
R² = 0.91995
Elysia timida 21°C
R² = 0.96643
Elysia timida 25°C
R² = 0.95487
21 days in starvation

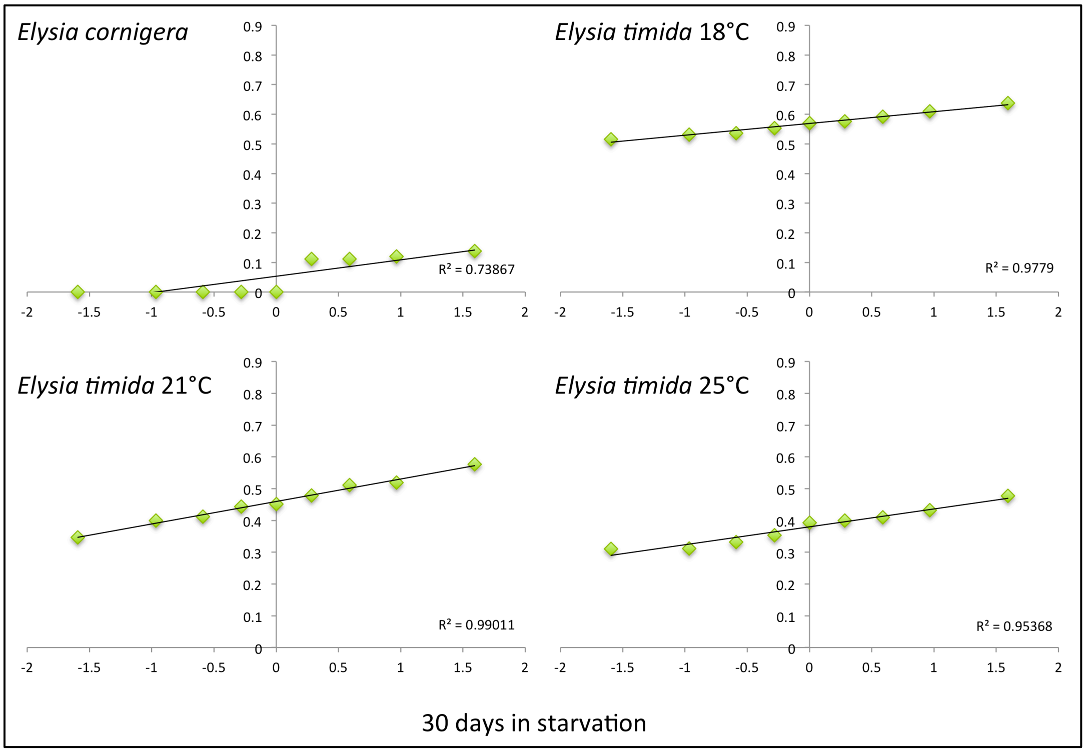

Interpretation: The recorded PAM values for each of the populations examined in these plots are assumed to have a normal distribution since the data points are best fit by straight lines and display r^2 values > 0.90. The only population that does not have an r^2 > 0.90 is *E. cornigera* starved for 30 days and this is likely due to a lack of measureable chlorophyll fluorescence observed in this species at this time point (a high number of zeros). Since the other populations do not display recordings in this range, *E. cornigera* was included to analyze this discrepancy.

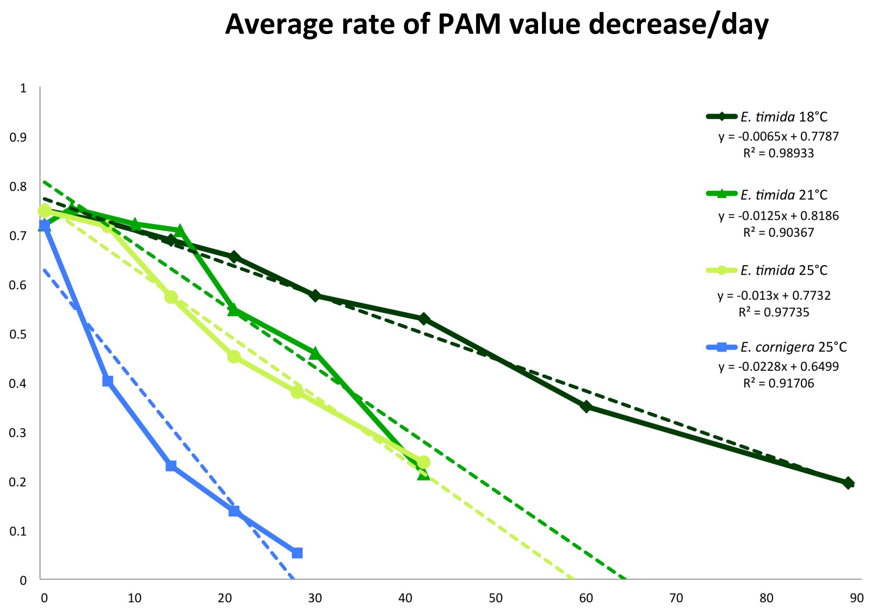

PAM - Levene's Test for Homogeneity of Variance:

Unstarved Specimens - Levene ANOVAs (PAM)				
	***E. timida* 18°C**	***E. timida* 21°C**	***E. timida* 25°C**	**E. cornigera 25°C**
**n**	10	10	10	10
**Sum**	0.305333	0.168889	0.373556	0.452268
**Mean**	0.033926	0.018765	0.041506	0.050252
**Sum of squares**	0.016858	0.003715	0.021498	0.035477
**Variance**	0.000812	0.000068	0.000749	0.001594
**Standard dev.**	0.028503	0.008258	0.027371	0.039921
**Standard error**	0.009501	0.002753	0.009124	0.013307
**Summary**		**Degrees of**		
	**Sum of Squares**	**Freedom**	**Mean Squares**	**F statistic: 1.99**
**Treatment**	0.004812	3	0.001604	**P value: 0.135**
**Error**	0.025788	36	0.000806	**not significant**

7 Days Starved Specimens - Levene ANOVAs (PAM)				
	***E. timida* 18°C**	***E. timida* 21°C**	***E. timida* 25°C**	**E. cornigera 25°C**
**n**	10	10	10	10
**Sum**	0.373	0.25	0.408	0.446987
**Mean**	0.041444	0.027778	0.045333	0.401903
**Sum of squares**	0.024293	0.01026	0.025976	0.038278
**Variance**	0.001104	0.000414	0.000935	0.00201
**Standard dev.**	0.033231	0.020358	0.030578	0.044831
**Standard error**	0.011077	0.006786	0.010193	0.014944
**Summary**		**Degrees of**		
	**Sum of Squares**	**Freedom**	**Mean Squares**	**F statistic: 4.86**
**Treatment**	0.894491	3	0.298164	**P value: <.0001**
**Error**	0.01963	36	0.000613	**significant**

14 Days Starved Specimens - Levene ANOVAs (PAM)				
	***E. timida* 18°C**	***E. timida* 21°C**	***E. timida* 25°C**	**E. cornigera 25°C**
**n**	10	10	10	10
**Sum**	0.419556	0.334222	0.631333	0.603432
**Mean**	0.046617	0.037136	0.070148	0.067048
**Sum of squares**	0.026648	0.02212	0.065941	0.055249
**Variance**	0.000886	0.001213	0.002707	0.001849
**Standard dev.**	0.029769	0.034835	0.052026	0.042998
**Standard error**	0.009923	0.011612	0.017342	0.014333
**Summary**		**Degrees of**		
	**Sum of Squares**	**Freedom**	**Mean Squares**	**F statistic: 1.38**
**Treatment**	0.006874	3	0.002291	**P value: <0.001**
**Error**	0.053242	36	0.001664	**significant**

21 Days Starved Specimens - Levene ANOVAs (PAM)				
	***E. timida* 18°C**	***E. timida* 21°C**	***E. timida* 25°C**	**E. cornigera 25°C**
**n**	10	10	10	10
**Sum**	0.530667	0.204	0.356444	0.158432
**Mean**	0.058963	0.022667	0.039605	0.017604
**Sum of squares**	0.050404	0.006494	0.018102	0.003188
**Variance**	0.002389	0.000234	0.000498	0.00005
**Standard dev.**	0.048881	0.015289	0.022319	0.007061
**Standard error**	0.016294	0.005096	0.00744	0.002354
**Summary**		**Degrees of**		
	**Sum of Squares**	**Freedom**	**Mean Squares**	**F statistic: 3.91**
**Treatment**	0.009449	3	0.00315	**P value: 0.0163**
**Error**	0.025369	36	0.000793	**significant**

30 Days Starved Specimens - Levene ANOVAs (PAM)				
	***E. timida* 18°C**	***E. timida* 21°C**	***E. timida* 25°C**	**E. cornigera 25°C**
**n**	10	10	10	10
**Sum**	0.280889	0.492667	0.423111	0.533333
**Mean**	0.03121	0.054741	0.047012	0.059259
**Sum of squares**	0.012619	0.039452	0.025961	0.032486
**Variance**	0.000482	0.00156	0.000759	0.00011
**Standard dev.**	0.021944	0.039502	0.027544	0.010494
**Standard error**	0.007315	0.013167	0.009181	0.003498
**Summary**		**Degrees of**		
	**Sum of Squares**	**Freedom**	**Mean Squares**	**F statistic: 1.88**
**Treatment**	1.794022	3	0.598007	**P value: <0.0001**
**Error**	0.032052	36	0.001002	**significant**

Interpretation: Examining the PAM values for each of the starvation time points reveals that the variance for each population after starvation begins (7, 14, 21, 30 days in starvation) cannot be considered homogenous due to the significant p values. Therefore, a standard ANOVA analysis cannot be performed and the Welch ANOVA is instead applied to compare these populations. Games-Howell tests were used following each Welch ANOVA as a post hoc analysis to compare between each group. This test was chosen due to the small sample size and the unequal variances revealed by the Levene's tests.

PAM - Welch ANOVAs and Games-Howell Post Hoc Analyses:

PAM values – 0 days in starvation				
	**E. timida 18°C**	**E. timida 21°C**	**E. timida 25°C**	**E. cornigera 25°C**
**mean**	0.749222	0.743556	0.770111	0.709778
**n**	9	9	9	9
**b**	0.068744885	0.079967989	0.051068959	0.083924451
**a**	0.002488343	0.090607579	3.118850435	5.012767931
**mean'**	3583.472343	2754.7282	5143.175166	2372.785522
**w**	4782.923193	3704.804812	6678.484561	3342.997761
**variance**	0.001881694	0.002429278	0.001347611	0.002692194
**sum of w**	18509.21033			
**sum of mean'/sum of w**	0.748500935		**p for lower df:**	0.090075705
**sum of a/k-1**	2.741571429		**p for higher df:**	0.088087596
**sum of b**	0.283706285			
	**degrees of freedom**		**F-statistic:**	**2.549**
**among groups**	3		**p-value:**	**0.088835402**
**within groups**	17.624		**p > 0.05**	**not significant**

Games-Howell results – 0 days in starvation			
E. timida 18°C	vs.	E. timida 21°C	not significant
E. timida 18°C	vs.	E. timida 25°C	not significant
E. timida 18°C	vs.	E. cornigera 25°C	not significant
E. timida 21°C	vs.	E. timida 25°C	not significant
E. timida 21°C	vs.	E. cornigera 25°C	not significant
E. timida 25°C	vs.	E. cornigera 25°C	not significant

PAM values – 7 days in starvation				
	**E. timida 18°C**	**E. timida 21°C**	**E. timida 25°C**	**E. cornigera 25°C**
**mean**	0.719444	0.753111	0.716333	0.401903
**n**	9	9	9	9
**b**	0.080347588	0.031999398	0.083139882	0.096087627
**a**	1.611361269	24.2774777	1.122137924	163.099252
**mean'**	2176.999085	5678.566442	2016.578042	755.9691118
**w**	3025.944674	7540.144287	2815.139193	1880.975606
**variance**	0.002974278	0.001193611	0.003197	0.004784751
**sum of w**	15262.20376			
**sum of mean'/sum of w**	0.696368155		**p for lower df:**	3.48213E-09
**sum of a/k-1**	63.37007629		**p for higher df:**	1.68817E-09
**sum of b**	0.291574495			
	**degrees of freedom**		**F-statistic:**	**58.798**
**among groups**	3		**p-value:**	**3.21613 E-09**
**within groups**	17.148		**p < 0.05**	**significant**

Games-Howell results – 7 days in starvation				
E. timida 18°C	vs.	E. timida 21°C	not significant	
E. timida 18°C	vs.	E. timida 25°C	not significant	
E. timida 18°C	vs.	E. cornigera 25°C	significant	p < 0.01
E. timida 21°C	vs.	E. timida 25°C	not significant	
E. timida 21°C	vs.	E. cornigera 25°C	significant	p < 0.01
E. timida 25°C	vs.	E. cornigera 25°C	significant	p < 0.01

PAM values – 14 days in starvation					
	**E. timida 18°C**	**E. timida 21°C**	**E. timida 25°C**	**E. cornigera 25°C**	
**mean**	0.690111	0.715444	0.569778	0.230044	
**n**	9	9	9	9	
**b**	0.061929735	0.038843052	0.096824336	0.0921431	
**a**	12.72202024	35.73783021	2.993039033	200.0323846	
**mean'**	1883.0564	2917.498395	629.4384398	299.7886917	
**w**	2728.627854	4077.882522	1104.70865	1303.17979	
**variance**	0.003298361	0.002207028	0.008146944	0.006906184	
**sum of w**	9214.398816				
**sum of mean'/sum of w**	0.621829166		**p for lower df:**	3.90582E-10	
**sum of a/k-1**	83.82842469		**p for higher df:**	1.6826E-10	
**sum of b**	0.289740222				
	**degrees of freedom**		**F-statistic:**	**77.816**	
**among groups**	3		**p-value:**	**3.33481E-10**	
**within groups**	17.257		**p < 0.05**	**significant**	

Games-Howell results – 14 days in starvation				
E. timida 18°C	vs.	E. timida 21°C	not significant	
E. timida 18°C	vs.	E. timida 25°C	significant	p < 0.05
E. timida 18°C	vs.	E. cornigera 25°C	significant	p < 0.01
E. timida 21°C	vs.	E. timida 25°C	significant	p < 0.01
E. timida 21°C	vs.	E. cornigera 25°C	significant	p < 0.01
E. timida 25°C	vs.	E. cornigera 25°C	significant	p < 0.01

PAM values – 21 days in starvation				
	**E. timida 18°C**	**E. timida 21°C**	**E. timida 25°C**	**E. cornigera 25°C**
**mean**	0.654444	0.548	0.451556	0.154224
**n**	9	9	9	9
**b**	0.116028823	0.064133901	0.100850277	0.022264844
**a**	165.086164	604.555943	74.72506829	580.1193524
**mean'**	934.8423192	6075.762242	1796.022588	3483.327151
**w**	1428.451761	11087.15738	3977.412227	22586.15489
**variance**	0.006300528	0.00081175	0.002262778	0.000398474
**sum of w**	39079.17625			
**sum of mean'/sum of w**	0.314488571		**p for lower df:**	1.42294E-15
**sum of a/k-1**	474.8288426		**p for higher df:**	2.66447E-16
**sum of b**	0.303277845			
	**degrees of freedom**		**F-statistic:**	**439.301**
**among groups**	3		**p-value:**	**8.6027E-16**
**within groups**	16.487		**p < 0.05**	**significant**

Games-Howell results – 21 days in starvation				
E. timida 18°C	vs.	E. timida 21°C	significant	p < 0.01
E. timida 18°C	vs.	E. timida 25°C	significant	p < 0.01
E. timida 18°C	vs.	E. cornigera 25°C	significant	p < 0.01
E. timida 21°C	vs.	E. timida 25°C	significant	p < 0.01
E. timida 21°C	vs.	E. cornigera 25°C	significant	p < 0.01
E. timida 25°C	vs.	E. cornigera 25°C	significant	p < 0.01

PAM values – 30 days in starvation					
	**E. timida 18°C**	**E. timida 21°C**	**E. timida 25°C**	**E. cornigera 25°C**	
**mean**	0.575778	0.459667	0.379889	0.053889	
**n**	9	9	9	9	
**b**	0.060592633	0.082936401	0.064472208	0.074312872	
**a**	115.1088724	11.71490056	0.00032363	238.9569985	
**mean'**	1721.197583	838.8928318	1053.58488	121.4211701	
**w**	2989.343544	1825.002535	2773.402725	2253.176353	
**variance**	0.003010694	0.0049315	0.003245111	0.003994361	
**sum of w**	9840.925157				
**sum of mean'/sum of w**	0.379547289		**p for lower df:**	1.92981E-11	
**sum of a/k-1**	121.9270317		**p for higher df:**	7.04057E-12	
**sum of b**	0.282314115				
	**degrees of freedom**		**F-statistic:**	**113.391**	
**among groups**	3		**p-value:**	**1.05858E-11**	
**within groups**	17.711		**p < 0.05**	**significant**	

Games-Howell results – 21 days in starvation				
E. timida 18°C	vs.	E. timida 21°C	significant	p < 0.01
E. timida 18°C	vs.	E. timida 25°C	significant	p < 0.01
E. timida 18°C	vs.	E. cornigera 25°C	significant	p < 0.01
E. timida 21°C	vs.	E. timida 25°C	significant	p < 0.01
E. timida 21°C	vs.	E. cornigera 25°C	significant	p < 0.01
E. timida 25°C	vs.	E. cornigera 25°C	significant	p < 0.01
